# Irisin Activates M1 Macrophage and Suppresses Th2-Type Immune Response in Rats with Pelvic Inflammatory Disease

**DOI:** 10.1155/2022/5215915

**Published:** 2022-02-10

**Authors:** Zhenge Zhang, Chongyuan Zhang, Shuirong Zhang

**Affiliations:** Department of Gynecology, Jingzhou Central Hospital, Jingzhou Hospital, Yangtze University, Jingzhou, China

## Abstract

**Objective:**

To investigate the mechanism of irisin to treat rats with acute pelvic inflammatory disease (APID).

**Methods:**

Female rats were established as APID. Firstly, the content of IL-6, IL-8, TNF-*α*, and NF-kB was tested in rats' serum by enzyme linked immunosorbent assay (ELISA). Interferon-*γ* (IFN-*γ*) and IL-4 in the supernatant of pelvic homogenates were also detected. The mRNA expression of the inducible nitric oxide synthase (iNOS), TNF-*α*, chemokine ligand 1 (CXCL1), arginase-1(Arg1), and chitinase-3-like-3 (Chi313) genes in the pelvic cavity was detected by quantitative reverse transcription polymerase chain reaction (RT-qPCR). IFN-*γ* and IL-4 secreted by spleen CD4^+^T cells and CD8^+^T cells were counted by flow cytometry, and the ratio of IFN-*γ*/IL-4 in CD4^+^T cells and CD8^+^T cells in the spleen was also detected by flow cytometry.

**Results:**

Irisin reduced the levels of IL-6, IL-8, TNF-*α*, and NF-kB in serum. Compared with the APID group, the expression level of IL-4 in the APID + Irisin group was reduced in the homogenate. At the same time, Irisin promotes the activation of M1 macrophages in the uterus, ovaries, and uterine tubes of rats with APID. Irisin also inhibited Th2-type immune response.

**Conclusions:**

Irisin activates M1 macrophage and suppresses Th2-type immune response in APID rats.

## 1. Introduction

Acute pelvic inflammatory disease (APID) is one of the gynecological diseases with high incidence and great harm in clinic. APID is a type of gynecological disease that refers to pelvic cavity reproductive organs such as the uterus, uterine tubes, and pelvic inflammation [1, 2]. The main reason is the anaerobic bacteria infection. People with poor hygiene during the menstrual period, irregular reproductive tract infections, sexual life, etc. are prone to anaerobic bacteria infection, which in turn causes APID [3]. Patients usually experience lower abdominal pain and increased leucorrhea, often accompanied by dizziness, high fever, loss of appetite, and other phenomena, and improper treatment may lead to chronic pelvic inflammatory disease, leading to pelvic effusion, causing pelvic adhesions, etc., which will have a greater impact on menstruation and pregnancy [4]. In addition, studies have confirmed that PID is associated with an increased risk of borderline ovarian tumors, particularly among women who had had multiple episodes of PID [5]. Therefore, APID should arouse our attention.

APID can induce innate immune response and adaptive immune response [6]. The former is the body's first line of defense against pathogen invasion and plays an important role in the early removal of pathogens and infection control. Among them, macrophages are the most important innate immune cells. Macrophages are dynamic heterogeneous cells with heterogeneity. Under different microenvironments and different inducing signals in the body, macrophages can change their morphology and physiological characteristics and differentiate into different activation states to deal with the external environment. According to the difference in the activation mode, phenotype, and secretion of cytokines of macrophages, macrophages can be divided into two types: classically activated macrophages (CAMS or M1) and alternatively activated macrophages (AAMs or M2). Under normal circumstances, in the process of inflammatory response, the balance of M1 and M2 macrophages is constantly changing due to the regulation of different cell signals [7, 8]. If the balance shifts to either side, the final outcome of the inflammatory response may be changed. Therefore, if the activation of M1 macrophages is promoted in the early stages of infection, it will help the body eliminate pathogens. At present, studies have confirmed that lipopolysaccharide, IFN-*γ,* and so on can activate M1. Also, IL-4, IL-10, IL-13, and so on can promote the activation of M2-type macrophages.

Although innate immunity can fight infection at an early stage, the body's complete and thorough elimination of pathogens mainly depends on adaptive immunity [9]. The main pathogen of APID is aerobic bacteria, followed by ureaplasma urealyticum, chlamydia trachomatis, and anaerobic bacteria. Studies have found that patients with impaired humoral immunity are prone to chronic infection after infection, indicating that antibodies have a certain role in preventing pathogen infection, but the protective effect is not complete [10]. Therefore, cellular immunity plays an important role in eliminating intracellular pathogens and preventing chronic and persistent infections. The cellular immune response is mediated by T lymphocytes [11], including CD4+T cells and CD8+T cells. CD4+T cells mainly include T-helper cell type 1 (Th1), Th2, Th3, and Th17 and regulatory T cells [12]. Th1 cells mainly secrete cytokines such as IFN-*γ*, TNF-*α,* and IL-2, which mediate cellular immune responses and play an important role in the complete elimination of pathogens [13]. If immune regulation can promote Th2 immunity to Th1-type immunity, it can promote the killing and elimination of pathogens by the body's immune system.

Irisin is a soluble peptide consisting of 112 amino acids [14]. Irisin promotes the browning of white fat by activating uncoupling protein 1 (UCP 1) to increase energy metabolism and is, therefore, considered to play a key role in metabolic diseases [14]. In recent years, more and more studies have shown that irisin has anti-inflammatory, antioxidative stress, and antiapoptotic effects in the physiological and pathological processes of lung injury and brain injury [15, 16]. Also, some studies have suggested that irisin and UCP2 work synergistically to improve alveolar epithelial cell damage in acute lung injury [17]. Irisin has different effects on the proliferation and apoptosis of tumor cells in breast cancer, lung cancer, and liver cancer through various mechanisms. We all know that proliferation and apoptosis of tumor cells are very important for tumor development, suggesting that irisin may have a similar effect with genitourinary cancers [18]. However, there is still a lack of research on the role of irisin in the pathophysiological process of APID.

This study intends to make a rat model of APID by injecting mixed bacteria into the uterus to study the anti-inflammatory mechanism of irisin on APID in rats and to provide a more adequate basis for the clinical application of irisin in the treatment of APID.

## 2. Materials and Methods

### 2.1. Animal

30 female SD rats, weighing 200–240 g and 8 weeks old, were provided by Beijing Weitonglihua Experimental Animal (Beijing, China). Five rats in each cage were raised in the Experimental Animal Center of Yangtze University. The rearing temperature was 20–25°C, and the humidity was 50%–70%. During the feeding period, they were given free access to food and drinking water. All animal feeding and experiment procedures meet the requirements of the Animal Experiment Ethics Committee of Yangtze University.

### 2.2. Bacteria Preparation

*Staphylococcus aureus* (strain number: 26001) and *Escherichia coli* (strain number: 44138), after biochemical identification, were cultured with ordinary meat intestine broth medium at 37°C, and 1 × 109 bacteria/mL were taken in the logarithmic growth stage and mixed in 1 : 1 for use. A special medium for mycoplasma was used, and *Ureaplasma urealyticum* was cultured in an 85% nitrogen-containing incubator. 1 × 106 pieces/mL were taken and mixed with the abovementioned mixed bacterial solution for modeling.

### 2.3. Construction of the APID Rat Model

Of the 30 rats, 10 rats were randomly selected as the sham operation group, and the remaining 20 rats were used for modeling and were randomly divided into the model group and irisin treatment group, with 10 rats in each group. Among them, rats in the irisin treatment group were injected intraperitoneally at a concentration of 100 *μ*g/kg irisin, once a day, for 14 consecutive days, and then, the model was made. Experimental rats were weighed, routinely disinfected, and anesthetized by intraperitoneal injection of 1% pentobarbital sodium (5 mg/100 g). After the anesthesia took effect, 0.2 mL of 3 billion/mL of mixed bacterial fluid was extracted. The needle of the syringe (the tip of the needle was cut off) was used to carefully enter one side of the uterine cavity at the bifurcation of the cervix at the bottom of the vagina. Then, the bacterial fluid was injected towards the ovary, and 0.1 mL was injected into each side of the uterus. After the injection, the absorbent gelatin sponge was placed on the cervix at the bottom of the vagina to prevent leakage of the medicine. In the sham operation group, the operation was the same as above, and the same amount of 0.9% saline was injected. After the operation, the animals were not given any treatment, drinking water was restored, and the animals were kept clean and kept for another 10 days.

### 2.4. Sample Collection

Rats anesthetized with 1% pentobarbital sodium (5 mg/100 g) were intraperitoneally injected and fixed on the operating table. A central abdominal incision was made to open the abdominal cavity and chest cavity. Blood samples were collected by rapid heart puncture in a 1.5 mL EP tube. At the same time, the uterus, ovaries, hoses, and spleen tissues of the rats were quickly removed. The collected tissue was placed in a 1.5 mL cryopreserved tube and stored in a refrigerator at −80°C for subsequent experiments.

### 2.5. Enzyme Linked Immunosorbent Assay (ELISA)

The rat pelvic tissue was homogenized and centrifuged at 12,000 rpm at 4°C for 5 min, and the supernatant was transferred to a new EP tube and stored at −80°C. Indirect ELISA (Yi Fei Xue, Nanjing, China) was used to detect the expression levels of IFN-*γ* and IL-4 in the uterus, ovaries, and transfusion tube homogenate supernatant. Besides, ELISA was also used to detect the expression levels of proinflammatory cytokines IL-6, IL-8, TNF-*α,* and NF-kB in the serum. We repeated all the experiments three times.

### 2.6. Quantitative Reverse Transcription Polymerase Chain Reaction (RT-qPCR)

About 20 mg of pelvic tissue was cut into an EP tube prefilled with 1 mL of TRIzoI reagent (R&D, Minneapolis, MN, USA), then a homogenizer was used to homogenize the tissue, and it was left for 5 min at room temperature and centrifuged at 4°C, 12,000 rpm for 10 min, and the supernatant was aspirated. Next, 0.2 mL of chloroform was added to the EP tube, shocked for 15 sec, allowed to stand at room temperature for 5 min, and centrifuged at 12,000 rpm for 15 min at 4°C. Next, the RNA in the water phase was transferred to a new EP tube. At the same time, 1 mL of isopropanol was added to it, pipetted to mix, and allowed to stand at room temperature for 10 min. The RNA pellet was centrifuged at 4°C at 12,000 rpm for 10 min, the supernatant discarded, and 1 mL of 75% ethanol was used to wash the RNA pellet. After centrifugation, the RNA pellet was dissolved with 50 *μ*L RNase-Free ddH2O. Next, the reverse transcription system mixture was configured according to the instructions, and reverse transcription was performed at 55°C for 15 minutes and 85°C for 5 seconds. Then, fluorescence staining RT-qPCR was used to detect the mRNA transcription level of macrophage activation marker genes. With GAPDH as the internal reference gene, the data were relatively quantitatively analyzed by the 2-ΔΔCt method. Primers are shown in Table 1. We repeated all the experiments three times.

### 2.7. IFN-*γ* and IL-4 were Detected by FCM in T Lymphocytes of Rat Spleen

The fresh spleen tissues of rats were placed in 5 mL EP tubes, 2 mL precooled Hank's solution (Camilo Biological, Nanjing, China) was added to it, homogenized by using a homogenizer, and centrifuged at 1200 rpm for 5 min, and the supernatant was discarded. Next, 3 mL erythrocyte lysate was used for resuspended precipitation, and it was allowed to stand for 5 min. Then, 5 mL PBS solution was used to stop the cleavage reaction and centrifuged at 4°C and 1200 rpm for 5 min, and the supernatant was discarded. 5 mL 1640 incomplete medium was used to add resuspended precipitation, which was centrifuged at 1200 rpm for 5 min at 4°C. The precipitation was retained and repeated to wash away the remaining red blood cell lysates. The cells were resuspended with 1640 complete medium (Thermo Fisher Scientific, Waltham, MA, USA), the cell density was adjusted to 2 × 106/100 *μ*L, and they were transferred to a 96-well plate with 100 *μ*L per well. After incubating the IL-4 and IFN-*γ* antibodies (Life Technology, China) at 4°C in the dark for 30 minutes, 300 *μ*L staining solution was used to resuspend the pellet. Flow cytometry was used to detect cytokines, and the data were analyzed using Flow Jo software. We repeated all the experiments three times.

### 2.8. Statistical Analysis

In this experiment, GraphPad Prism 8.0 software (La Jolla, CA, USA) was used to analyze the experimental data by one-way ANOVA and the Duncan test, and the experimental data were expressed as mean ± standard deviation (‾*X*±SD). *P* < 0.05 indicates a significant difference.

## 3. Results

### 3.1. Effect of Irisin on Rat Serum Cytokines

**Figure 1:**
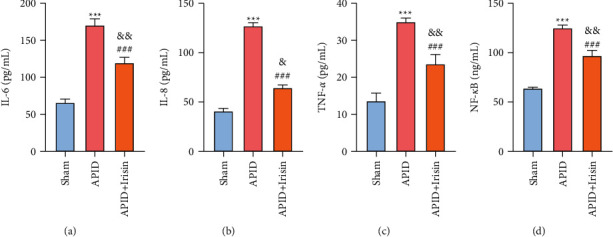
Effect of irisin on rat serum cytokines. (a ∼ d) Levels of IL-6, IL-8, TNF-*α*, and NF-kB in the rat serum were determine by ELISA (“^*∗∗∗*^” indicates a statistical difference from the sham group, *P* < 0.001; “###” indicates a statistical difference from the APID group, *P* < 0.001. “&” indicates a statistical difference from the sham group, *P* < 0.05; “&&” indicates a statistical difference from the sham group, *P* < 0.01).

In order to detect the level of inflammation in rats, we drew rat serum and used ELISA to detect the level of cytokines of rats. The results showed that compared with the sham group, the levels of IL-6, IL-8, TNF-*α*, and NF-kB in the APID group were increased, and the difference was statistically significant, suggesting that the rat model of APID was successfully made. Compared with the APID group, the content of IL-6, IL-8, TNF-*α*, and NF-kB in the irisin treatment group was reduced, and the difference was statistically significant (Figures 1(a)–1(d)), indicating that irisin treatment has a curative effect on the rat model of APID. However, irisin cannot completely restore IL-6, IL-8, TNF-*α*, and NF-kB levels. The abovementioned results indicate that irisin can reduce the level of inflammation in rats with APID.

### 3.2. Changes of IL-4 and IFN-*γ* Expression in Rat Uterus, Ovaries, and Uterine Tube Homogenates

**Figure 2:**
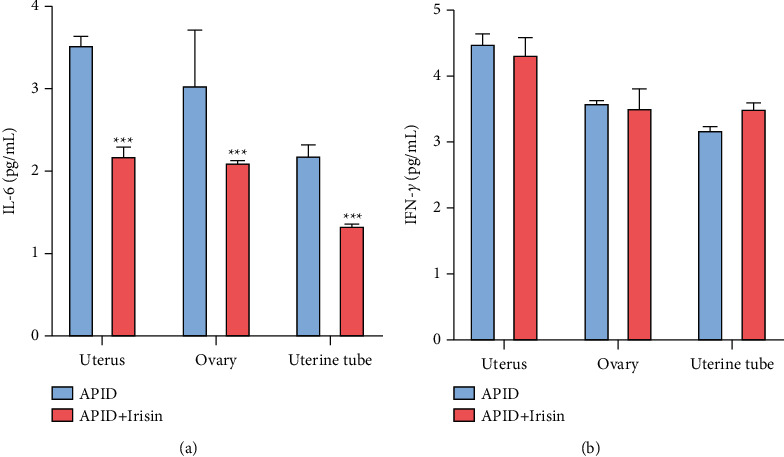
Changes of IL-4 and IFN-*γ* expression in rat uterus, ovaries, and uterine tube homogenates. (a), (b) Levels of IL-4 and IFN-*γ* in rat uterus, ovaries, and uterine tube were determine by ELISA (“^*∗∗∗*^” indicates a statistical difference from the APID group, *P* < 0.001).

The indirect ELISA method was used to detect the expression levels of IL-4 and IFN-*γ* in the supernatant of the uterus, ovaries, and uterine tube tissues on the 10th day of the model. Compared with the sham group, the expression level of IL-4 in the APID + irisin group was reduced in the homogenate (Figure 2(a)). However, there was no significant difference in the expression level of IFN-*γ* in the two groups of tissues (Figure 2(b)).

### 3.3. Level Detection of Macrophage Activation Marker Genes

**Figure 3:**
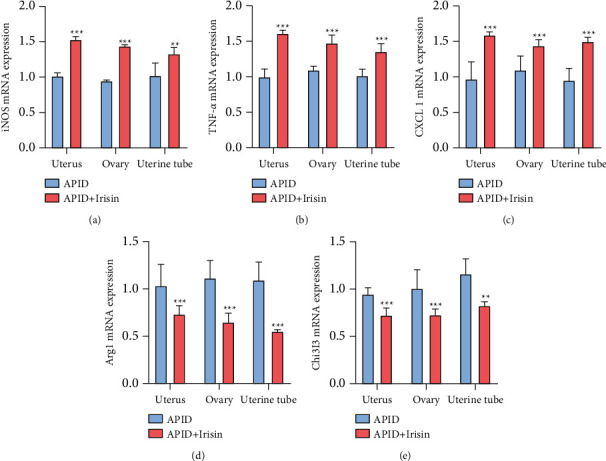
Level detection of macrophage activation marker genes. (a ∼ e) The mRNA expressions of iNOS, TNF-*α*, CXCL1, Arg1, and Chi3l3 in rat uterus, ovaries, and uterine tube were determined by RT-qPCR (“^*∗∗∗*^” indicates a statistical difference from the APID group, *P* < 0.001).

RT-qPCR detection showed that the relative transcription level of M1 macrophage activation marker iNOS gene mRNA in the uterus, ovaries, and uterine tube homogenates in the APID + irisin group was dramatically higher than that in the APID group. The relative transcription level of TNF-*α* and CXCL 1 gene mRNA was also higher than that of APID group (Figures 3(a)–3(c)), while the relative expression level of M2 macrophage activation marker Arg 1 gene mRNA was lower than that of APID group. At the same time, the relative transcription of Chi3l3 gene mRNA was also dramatically lower than that of the APID group (Figures 3(d) and 3(e)). The abovementioned results indicate that irisin promotes the activation of M1 macrophages in the uterus, ovaries, and uterine tube of rats with APID.

### 3.4. Irisin Inhibits Th2-Type Immunity in Rats with APID

**Figure 4:**
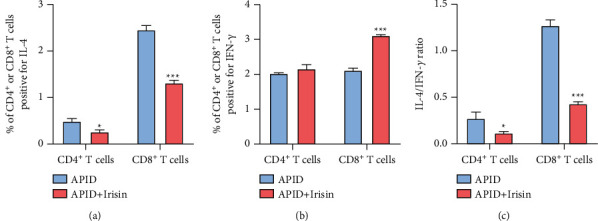
Irisin inhibits Th2-type immunity in rats with APID. (a, b) IFN-*γ* and IL-4 were detected by FCM in T lymphocytes of rat spleen (“^*∗*^” indicates a statistical difference from the APID group, *P* < 0.05; “^*∗∗∗*^” indicates a statistical difference from the APID group, *P* < 0.001).

On the 10th day after the infection of rats with APID, the ratio of CD4^+^T cells secreting IL-4 in the spleen of rats in the APDI + irisin group was lower than that in the APID group and the ratio of CD8^+^T cells secreting IL-4 in the spleen of rats in the APDI + irisin group was lower than that in the APID group (Figure 4(a)), while the ratio of CD8^+^T cells secreting IFN-*γ* in the spleen of rats in the APDI + irisin group was higher than that in the APID group. However, there was no significant difference in IFN-*γ* secreted by CD4^+^ T between the two groups (Figure 4(b)). Next, the rat spleen was taken to prepare lymphocytes, and the ratio of IL-4 to IFN-*γ* in CD4+ T cells and CD8+ T cells was detected by flow cytometry. The results showed that compared with the APID group, the ratio of IL-4/IFN-*γ* in CD4+ T cells of the APID + irisin group was lower and the ratio of IL-4/IFN-y in CD8+ T cells of the APID + irisin group was also lower than that of the APID group (Figure 4(c)). The abovementioned results indicate that irisin inhibits Th2-type immunity in APID rats.

## 4. Discussion

The results of this experiment show that irisin can promote the clearance of acute pelvic inflammatory pathogens in rats and reduce the inflammation of rat pelvic tissue caused by infection. At the same time, irisin can promote the activation of M1 macrophages and inhibit Th2 immunity in infected rats.

At present, most studies believe that APID is mostly a mixed infection, and it is caused by two sources: (1) endogenous pathogen, from the original bacteria residing in the vagina; (2) exogenous pathogen, mainly the pathogen of sexually transmitted diseases. However, the clinical treatment is mostly the combined application of antibiotics, and the use of sensitive antibiotics is the key to treatment. However, there is currently no better method other than antibiotics for treating APID. Long-term use of antibiotics can easily lead to the production of drug-resistant bacteria and double infections, resulting in prolonged illness and repeated attacks. Studies have shown that, during acute lung injury, irisin protects alveolar epithelial cells from damage by maintaining mitochondrial function and improving oxidative stress [19]. In addition, in the research of brain injury and type 2 diabetes, the effects on antioxidative stress, antiapoptosis, and anti-inflammatory are also described [20, 21]. In this study, we first detected serum cytokine levels and found that irisin dramatically reduced the level of inflammation in rats with APID.

Phagocytes are the main cells involved in the innate immune response and play an important role in the early killing and elimination of pathogens. We found that compared with the APID group, the expression level of IL-4 in the pelvic tissue of the irisin group was reduced, and there was no significant difference in the expression level of IFN-*γ*. The mRNA transcription level of M1 macrophage activation marker iNOS increased, and the mRNA transcription levels of TNF-*α* and CXCL 1 genes also increased, while the mRNA transcription levels of M2 macrophage activation markers Arg 1 and Chi3l3 decreased. The abovementioned results indicate that irisin can reduce the expression of IL-4 in the pelvic tissues of rats after infection, promote the activation of M1 macrophages after infection, and weaken the activation of M2 macrophages.

We also demonstrated that irisin can inhibit the infection of Th2-type immunity. The reduction and inhibition of the differentiation of Th2 cells were seen [22]. Moreover, when the expression of uncleared pathogen antigens in pelvic tissues is reduced, Th1-biased immunity will continue to be induced, and the positive feedback regulation formed will continue to increase the clearance of pathogens and shorten the course of infection.

## 5. Conclusions

This article explores the effect of irisin on APID and preliminarily explores the remote regulation mechanism of irisin on macrophage activation and specific immunity after infection, which will help to understand the host's regulation and mechanism of inflammation caused by infection.

## Figures and Tables

**Table 1:** Real-time PCR primers.

Gene name	Forward (5ʹ>3ʹ)	Reverse (5ʹ>3ʹ)
iNOS	GTTCTCAGCCCAACAATACAAGA	GTGGACGGGTCGATGTCAC
TNF-*α*	CCCTCACACTCAGATCATCTTCT	GCTACGACGTGGGCTACAG
CXCL1	CTGGGATTCACCTCAAGAACATC	CAGGGTCAAGGCAAGCCTC
Arg1	CTCCAAGCCAAAGTCCTTAGAG	AGGAGCTGTCATTAGGGACATC
Chi313	CAGGTCTGGCAATTCTTCTGAA	GTCTTGCTCATGTGTGTAAGTGA
GAPDH	GGAGCGAGATCCCTCCAAAAT	GGCTGTTGTCATACTTCTCATGG

## Data Availability

The datasets used and analyzed during the current study are available from the corresponding author on reasonable request.
